# Translation of 3D Anatomy to 2D Radiographic Angle Measurements in the Ankle Joint: Validity and Reliability

**DOI:** 10.1177/24730114221112945

**Published:** 2022-07-21

**Authors:** Gwendolyn Vuurberg, Nazli Tümer, Inger Sierevelt, Johannes G. G. Dobbe, Robert Hemke, Jan Joost Wiegerinck, Mario Maas, Gino M. M. J. Kerkhoffs, Gabriëlle J. M. Tuijthof

**Affiliations:** 1 Department of Orthopedic Surgery, Amsterdam Movement Sciences, Amsterdam UMC, University of Amsterdam, Amsterdam, the Netherlands; 2 Academic Center for Evidence-based Sports Medicine (ACES), Amsterdam, the Netherlands; 3 Amsterdam Collaboration on Health & Safety in Sports (ACHSS), AMC/VUmc IOC Research Center, Amsterdam, the Netherlands; 4 Radiology and Nuclear Medicine, Amsterdam Movement Sciences, Amsterdam UMC, University of Amsterdam, Amsterdam, the Netherlands; 5 Department of Radiology and Nuclear Medicine, Rijnstate Hospital, Arnhem, the Netherlands; 6 Department of Biomechanical Engineering, Delft University of Technology (TU Delft), the Netherlands; 7 Specialized Centre for Orthopedic Research and Education (SCORE), Xpert Orthopedics, Amsterdam, the Netherlands; 8 Spaarne Gasthuis Academy, Orthopedic Department, Hoofddorp; 9 Department of Biomedical Engineering and Physics, Amsterdam Movement Sciences, Amsterdam UMC, University of Amsterdam, Amsterdam, the Netherlands; 10 Bergman Clinics, Rijswijk, the Netherlands; 11 Department of Research Engineering, Faculty Health, Medicine & Life Science, Maastricht University, Maastricht, the Netherlands

**Keywords:** prognostic value, bone geometry, radiographic parameters, reliability analysis, chronic ankle instability

## Abstract

**Background:**

The objective consisted of 2 elements, primarily to define 2 bone geometry variations of the ankle that may be of prognostic value on ankle instability and secondly to translate these bone variations from a 3D model to a simple 2D radiographic measurement for clinical use.

**Methods:**

The 3D tibial and talar shape differences derived from earlier studies were translated to two 2D radiographic parameters: the medial malleolar height angle (MMHA) and talar convexity angle (TCA) respectively to ensure clinical use. To assess validity, the MMHA and TCA were measured on 3D polygons derived from lower leg computed tomographic (CT) scans and 2D digitally reconstructed radiographs (DRRs) of these polygons. To assess reliability, the MMHA and TCA were measured on standard radiographs by 2 observers calculating the intraclass correlation coefficient (ICC).

**Results:**

The 3D angle measurements on the polygons showed substantial to excellent agreement with the 2D measurements on DRR for both the MMHA (ICC 0.84-0.93) and TCA (ICC 0.88-0.96). The interobserver reliability was moderate with an ICC of 0.58 and an ICC of 0.64 for both the MMHA and TCA, respectively. The intraobserver reliability was excellent with an ICC of 0.96 and 0.97 for the MMHA and the TCA, respectively.

**Conclusion:**

Two newly defined radiographic parameters (MMHA and TCA) are valid and can be assessed with excellent intraobserver reliability on standard radiographs. The interobserver reliability was moderate and indicates training is required to ensure uniformity in measurement technique. The current method may be used to translate more variations in bone shape prior to implementation in clinical practice.

**Level of Evidence:**

Level III, cohort study.

## Introduction

Doherty et al^11^ reported a prevalence of ankle sprains of ±11% in both men and women and an incidence up to 7 ankle sprains per 1000 exposures in sports. At least one-third of individuals sustaining lateral ankle sprains in sports and recreational activities develop chronic ankle instability (CAI).^14,23^ A step in optimizing treatment and prevention strategies of ankle sprains and CAI is the identification of risk parameters for the onset of lateral ankle sprains and of CAI. This way patients who benefit from specific prevention programs or require early surgical stabilization may be identified at an early stage.

Intrinsic elements (individual related), like ligament laxity, neuromuscular control, hindfoot alignment, and osseous joint configuration, and extrinsic elements (environment related) such as injury mechanism are currently known risk factors for CAI.^5,6,12,13,17-19,21,27,31,33^ Although many modifiable risk parameters such as type of sports have been identified, a more careful analysis of bone morphology on top of hindfoot alignment has not been performed. Recent, more advanced methods could contribute to risk assessment as it enables the inclusion of bone geometry in a nonmodifiable factor. In this bone shape models provide a solution, defining the mean bone shape within a patient group and with that identifying bone shape differences compared to other populations or patient groups.

In a recent study by Tümer et al,^28^ 3D statistical shape models (SSMs) of the distal tibia and talus were built comparing healthy controls and patients with a talar osteochondral defect (OCD). A quantitative comparison of the bone shape variations between these 2 groups gave 2 shape models that were significantly different.^28^ These results are interesting in the light of CAI, because OCDs are often observed in combination with ankle instability and both entities share a common trauma mechanism. The inversion trauma of an ankle sprain causes excessive compression and shear force that occurs between the talus and tibia damaging the cartilage and ligaments.^1,2,20,22,30^

For this reason, we suspect similar bone shape differences may exist between patients experiencing CAI and OCDs. Also, the shape variations observed in the previously mentioned study influence ankle joint congruency and may alter the mechanical environment of the joint.^28^

Although 3D SSM is a powerful technique to describe complex geometries and shape variations within a studied population, its implementation in clinical settings has not been realized yet as there is no translation to conventional assessment methods such as radiographic evaluation. Therefore, the objective of this study is to firstly translate the 2 shape models derived from the SSMs into angles fit for plain radiographic assessment. Secondly, we assess the validity and reliability of these same radiographic parameters.^28^ This validation will result in 2 additional radiographic parameters that might be prognostic for the onset of lateral ankle sprains and CAI. We hypothesize that measurement of the parameters on a 3D model and 2D reconstructed radiographs will result in comparable results and therefore prove valid in 2D, which may subsequently be reliably measured on radiographs derived from a clinical setting.

## Materials and Methods

### Study Population

The data used in this study were derived from 2 study populations. The first constituted of prospectively collected data of healthy volunteers who had undergone a simulated weight-bearing CT scan for research purposes on lower leg bone symmetry to translate the shape models from 3D to 2D measurements.^32^ For this, a feasibility sample size of 20 participants was used in order to minimize radiation load in combination with maximum yield.

The ensure reliability of the measurements with the variations of radiographs in a clinical setting, the second part included retrospectively collected data of all subsequent patients aged 16 years or older visiting the ER department of Academic Medical Center (AMC, Amsterdam, The Netherlands) between October 2016 and October 2017. To ensure valid conclusions on the reliability of the 2 parameter measurements, a sample size of 50 was chosen.^26^

Patients of the second cohort were included when they visited the ER not more than 1 week after an ankle sprain, reported lateral ankle pain after a sprain or distortion, and of whom both a mortise view and lateral radiograph had been made were included in the reliability assessment. Exclusion criteria were a fracture or other intra-articular joint pathology (fracture/osteoarthritis), a diagnosed OCD after primary inclusion, medial ankle instability (ie, pain and the feeling of giving way primarily medially),^15^ previous ankle surgery, or an unreliable radiograph due to deviation of the projection angle blocking radiographic assessment of the angles. All patients were asked for informed consent. This study was approved by the local Internal Review Board (IRB) and was executed according to the local ethical standards and Declaration of Helsinki.

Terminology

*- 3D space angle*: an angle between 2 lines drawn on a polygon in 3D space;
*- 2D plane angle*: an angle between 2 lines on a polygon projected in a simulated 2D space (viewing plane);
*- Digitally reconstructed radiograph (DRR) angle*: an angle between 2 lines as measured on a 2D reconstructed radiograph based on volume rendering;
*- Radiograph*: mortise view and standard lateral radiograph.

### Image Acquisition and Bone Model Creation

For the first part of this study, a data set of simulated weightbearing CT scans were used. These included a bilateral CT scan of each volunteer of the knees, lower leg, and feet using a Brilliance 64 CT scanner (Philips, Amsterdam, the Netherlands) (voxel size 0.46 × 0.46 × 0.45 mm, 120 kV, and 160 mAs).^9^ Subsequently, of all CT scans, both the tibiae and tali were segmented in a custom-made software program by a threshold-connected region growing followed by a binary closing algorithm for filling of residual holes and closing of the outline.^10^ A Laplacian level-set segmentation growth algorithm was used to grow toward the cortical boundary of the bone. The Marching cubes algorithm was then used to extract a polygon mesh at the zero level of the level-set image, representing the virtual 3D surface models of the bones. All of the image analysis steps in this cohort were performed using custom-made software.^10^

The second set of data included standard mortise and lateral radiographs of the ankle joint as reference for the digitally reconstructed radiographs. The measurements were performed using the local PACS viewing software used by our radiology department.

### Translation of the 3D Bone Shape Variations Into 2D Radiographic Parameters

**Figure 1.**
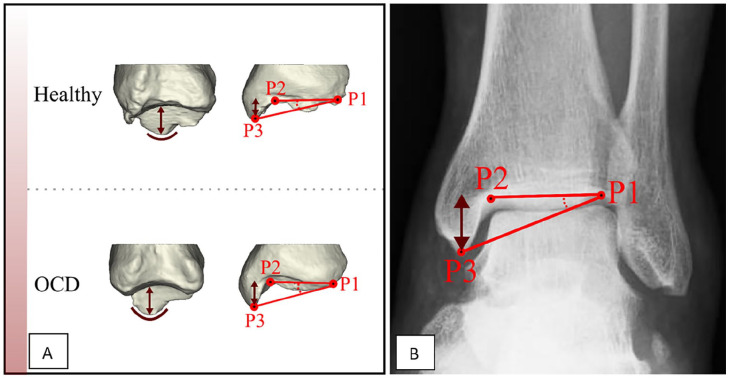
(A) The medial malleolus of patients with an osteochondral defect (OCD) showed differences with respect to those of controls (greater height and pointier aspect). (B) The medial malleolar height angle (MMHA) is defined on a mortise view by drawing 2 lines that pass from the distal tibial joint line P1-P2 and P1-P3. P1 is the most lateral point of the distal tibial joint line. P3 is the lowest point of the medial malleolus. The angle between the lines represents the MMHA.

In the previous study, the first shape model indicated differences in the medial malleolar height (which was combined with greater pointiness of the malleolus tip) between patients with an OCD and controls (Figure 1A).^28^ Based on this information, we defined the medial malleolar height angle (MMHA) on 3D as the angle between (1) the middle of the medial distal tibial joint surface to the middle of the lateral distal tibial joint surface and (2) the medial distal tibial joint surface and the most distal tip of the medial malleolus; and on a mortise radiograph as the angle between (1) the distal tibia surface and (2) the tip of the medial malleolus to be measured on a mortise view (Figure 1B). By choosing an angle instead of absolute height, we were less dependent on scaling issues.

**Figure 2.**
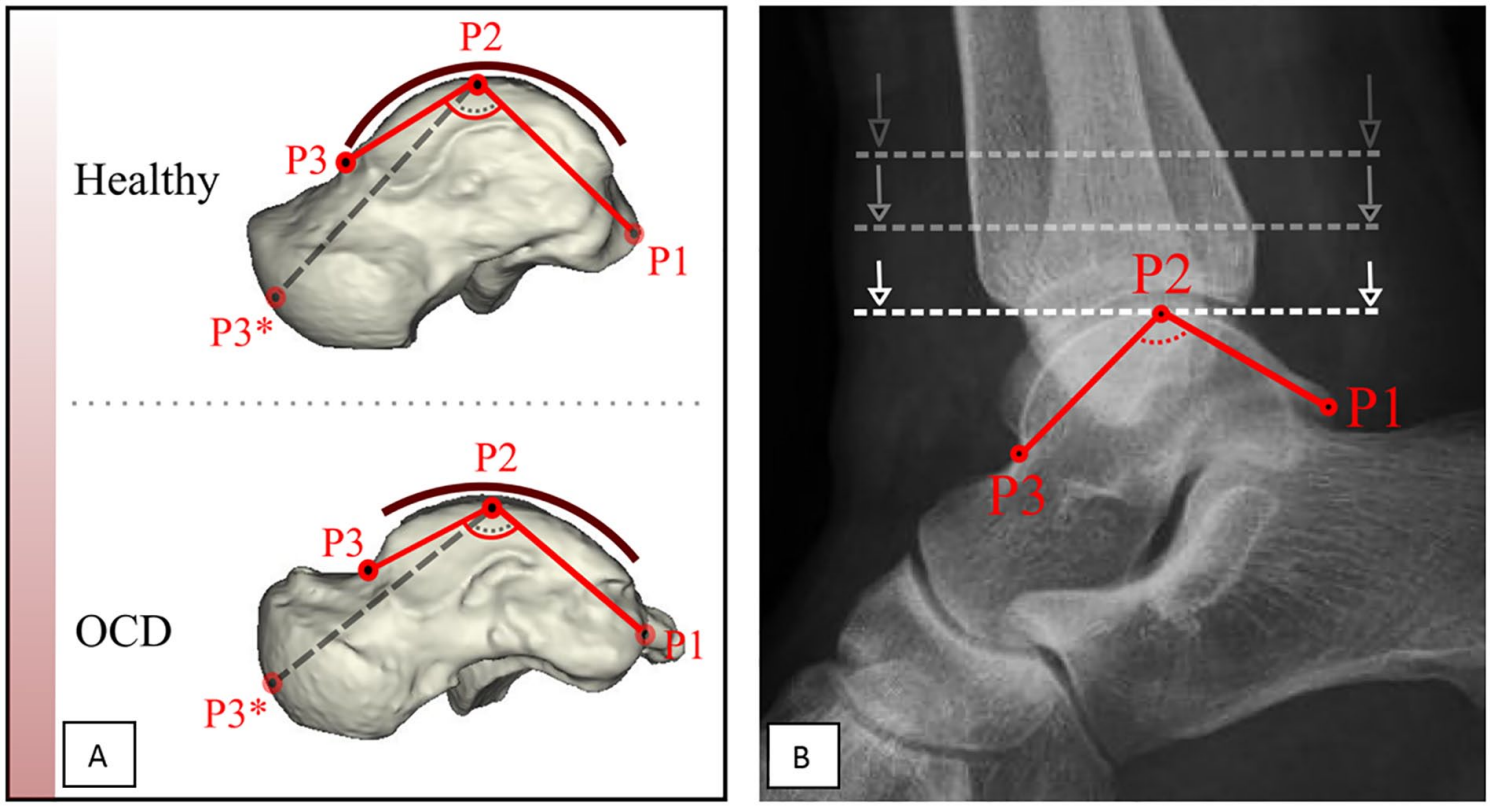
(A) The talar convexity in the osteochondral defect (OCD) group was significantly greater compared with controls. To ensure correct measurement on a lateral radiograph, as the anterior articular surface area of the talus is not always fully visible, it was decided to move P3* from the articular talar surface of the talonavicular joint to the talar neck to form P3. (B) The talar convexity angle (TCA): P1 represents the posterior tubercle of the talus. P2 is marked at the location where the line dropping down intersects the talus for the first time. P3 lies at the transition region of talar body to talar neck. P3 will be at the deepest point of the transition. The angle between the lines P1-P2 and P2-P3 is recorded as the TCA.

The second shape model described by Tümer et al^28^ mainly depicted changes in talar convexity (Figure 2A). Based on these differences, the talar convexity angle (TCA) was defined to be measured on both 3D and a lateral radiograph: the angle between 2 lines extending from the summit of trochlear surface to the midpoint of the convexity of the articular talar surface of the talonavicular joint and to the posterior tubercle of the talus (Figure 2B).

### Validity

As these shape differences measured using SSMs were originally defined in a 3D plane, the first step was to validate the new measurement parameters.

**Figure 3.**
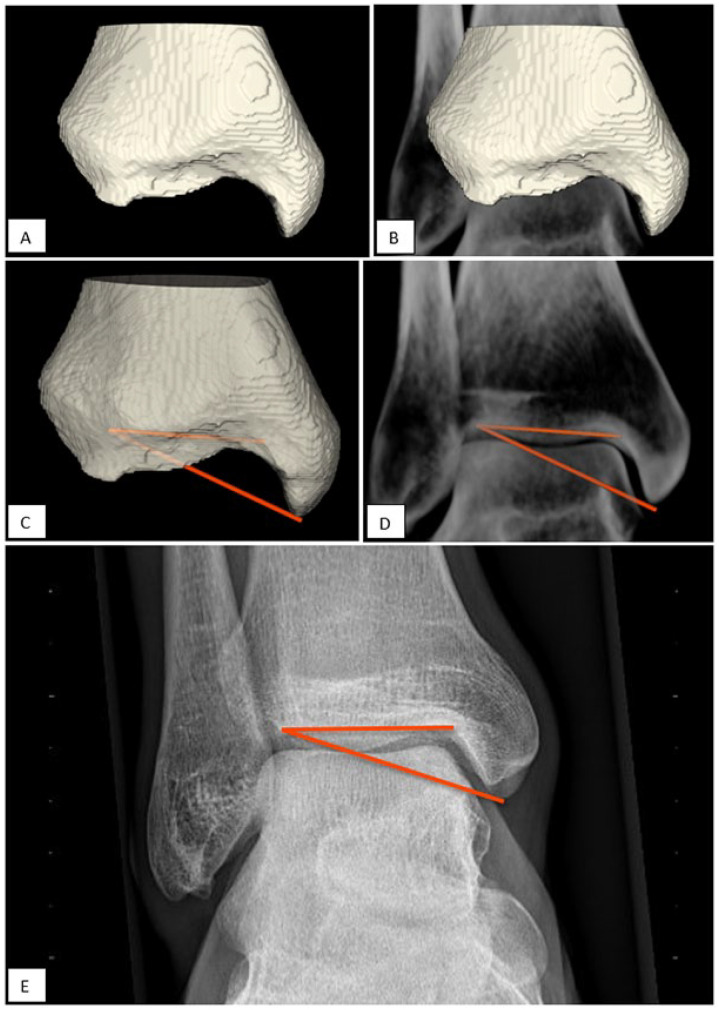
Validation of the MMHA. (A) 3D polygon based on computed tomographic segmentation. (B) 3D polygon included in the DRR. (C) MMHA measured from 2 lines defined in the 3D space. (D) MMHA measured on the DRR in a 2D plane. (E) MMHA measured on a mortise view. DRR, digitally reconstructed radiograph; MMHA, medial malleolar height angle.

**Figure 4.**
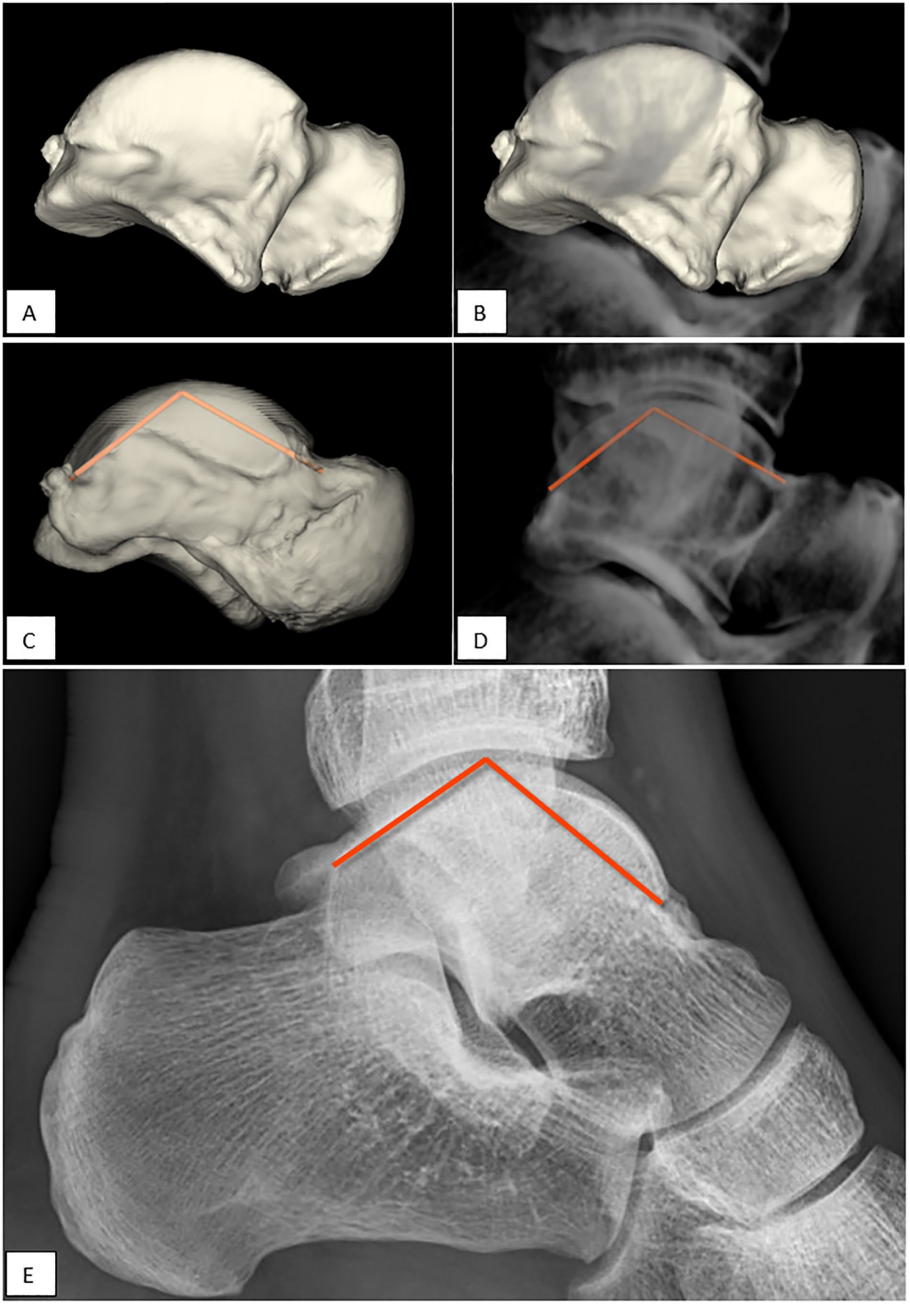
Validation of the TCA. (A) 3D polygon based on computed tomographic segmentation. (B) 3D polygon included in the DRR. (C) TCA measured between 2 lines in 3D space. (D) TCA measured on the DRR in 2D plane. (E) TCA measured on a standard lateral radiograph. DRR, digitally reconstructed radiograph; TCA, talar convexity angle.

First the MMHA and TCA were twice manually drawn by an experienced researcher (G.V.) in a 3D polygon with the help of 3D analysis software using a preexisting database of 20 lower leg simulated weightbearing CT scans (see Figures 3 and 4).^9^ The software determined the angle between the lines in 3D space (see Figures 3A-C and 4A-C). The MMHA indicated an ICC of 0.92 (0.86-0.96) and the TCA an ICC of 0.98 (0.96-0.99). Based on excellent reliability between the drawn lines, the last angle measurement was used for analysis.

To determine the same angle in the 2D plane, an anatomical coordinate system was used, which was automatically assigned based on the polygon mesh. The Z axis was along the direction of the tibia bone axis with the smallest eigenvalue. The X axis was placed perpendicular to the Z axis through the center of the medial malleolus. The Y axis was subsequently placed perpendicular to the X and Z axes.^7^ The polygon was aligned along the X axis for the MMHA and Y axis for the TCA, so the plane of view simulated a mortise and lateral radiograph for the MMHA and TCA, respectively (see Figures 3C and 4C).

**Figure 5.**
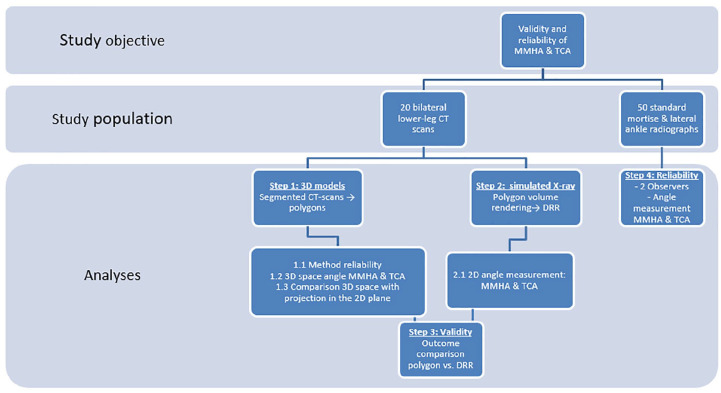
Flow chart of the study methodology.

Secondly, the angle between the lines in the 2D (viewing) plane was determined for both the MMHA and TCA. Thirdly, we created DRRs by volume rendering of the image volume according to the acquisition plane of regular radiographs to simulate a mortise and lateral radiographs and measured the MMHA and TCA manually (see Figures 3D and 4D). This was done because the volunteers for whom the CT scans were derived, no regular (clinical) radiographs were available. The determined angles in the 3D space, 2D plane, and as measured on the DRR were compared to define whether the translation from 3D anatomy to the 2D measurement was valid (see Statistical Analysis and the flow chart in Figure 5).

### Intra- and Interobserver Reliability

For angle measurement on the polygons (3D), the measurements of the MMHA and TCA were performed twice at least 1 week apart and assessed on intraobserver reliability before continuing to the measurement on DRR or conventional radiographs.

Reliability of the radiographic (2D) assessment in a clinical setting was assessed by 2 independent observers on ankle radiographs derived from the ER cohort. The 2 observers with both different levels of experience in radiographic assessment, a radiology resident and an orthopaedic resident, measured the angles on mortise views and standard lateral radiographs on the same set of radiographs (see Figures 3E and 4E). Observers were not aware of patient characteristics (eg, age, gender, and indication for radiographs).

To minimize effects of a potential learning curve on the results, a practice set of 10 cases was provided, which were not included in the analysis. The intraobserver reliability was assessed based on the measurements of the radiology resident, who measured all angles twice with a minimum time interval of 2 weeks in between the measurements. The second set of MMHA and TCA measurements acquired by the radiology resident were used to assess the intraobserver reliability.

### Statistical Analysis

Data distribution (normality vs skewed) was assessed using the Shapiro-Wilks test. Normally distributed data were presented with mean and SD. The validity and reliability of data were both reported using the intraclass correlation coefficient (ICC) with accompanying 95% confidence interval (95% CI). Interpretation of the ICC was as follows: ≤0.40, poor reliability; 0.40-0.75, moderate reliability; 0.75-0.90, substantial reliability; and >0.90, excellent reliability.^26^

With the future objective to use the current measurements in a risk assessment model, reliability is of utmost importance to avoid coincidental findings and minimize patient load when included in a study. For this reason, additionally, a cutoff value for the risk assessment model was defined. An ICC of 0.70 was deemed sufficient to reliably assess the correlation of a measurement with CAI. In addition to the ICC, reliability and variability in measured angles were visualized using Bland-Altman plots, and calculating the standard error of measurement (SEM) [SD√(1 – ICC)] and the minimal detectable difference (MDD) (1.96 × √2 × SEM).^4,8,26^

For the ICC, a *P* value of <.05 was regarded statistically significant. All statistical analyses were performed using SPSS 23.0 and IBM SPSS 22.0 (IBM Corp, Armonk, NY).

## Results

### Validity of the MMHA and TCA

The set of CT scans included data of 20 healthy volunteers (10 men, 10 women) with a mean (SD) age of 37.7 (±11.1) years for men and 34.0 (±10.3) years for women. Men had a mean weight of 82.7 (±5.6) kg and body height of 185 (±5) cm. Women reported a mean weight of 71.0 (±11.6) kg and a mean body height of 173 (±8) cm.

**Figure 6.**
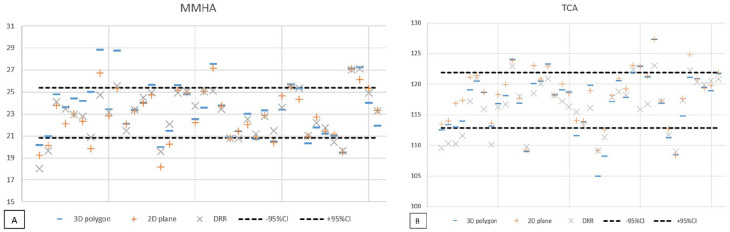
Plot of the (A) MMHA and (B) TCA measurement on a 3D polygon, 2D plane simulation and digitally reconstructed radiograph. MMHA, medial malleolar height angle; TCA, talar convexity angle.

The MMHA was larger when measured in 3D space than in the 2D planes with a mean difference (MD) of 0.51 degrees (SD ± 1.26; *P* = .014) (see Figure 6A). There was no significant difference between the measurement of the MMHA in 3D space and on DRR (MD = 0.43; SD ± 1.3) or between the measurement in the 2D plane and on DRR (MD = −0.08; SD ± 0.84).

For the TCA, greater angles were measured in 3D space and 2D plane compared to the measurements on the DRR (see Figure 6B). Although small, all measurements showed significant differences between imaging planes, the 3D plane vs the 2D plane showed an MD of –1.07 degrees (SD ±1.38; *P* ≤ .005); the 3D plane vs the DRR, MD = 0.81 (SD ±2.32; *P* = .033) and the 2D plane vs the DRR, MD = 1.88 (SD ±2.09; *P* < .005).

Despite these differences in absolute angle measurements of the MMHA and TCA, comparing these angles in 3D space, 2D plane, and DRR showed substantial to excellent agreement. When translated from the 3D shape model to the simulated mortise view and lateral radiograph without any outliers per step in the translation (MMHA ICC: 0.84-0.93; TCA ICC: 0.88-0.97). As the correlation coefficient was not affected by translation to the 2D plane, Table 1 shows the ICC of the translation from the 3D plane to DRR.

**Table 1.** Validity of the MMHA and TCA: Agreement Between the Angle Measurement in 3D Space to the 2D Measurement on DRR.

3D Space vs DRR	MMHA	TCA
ICC (95% CI)	0.84 (0.72-0.91)	0.97 (0.77-0.93)
SEM	0.91	1.62
MDD	2.65	3.53

Abbreviations: DRR, digitally reconstructed radiograph; ICC, intraclass correlation coefficient; MDD, minimal detectable difference; MMHA, medial malleolar height angle; SEM, standard error of measurement; TCA, talar convexity angle.

### Reliability of the MMHA and TCA

Of the total of 50 patients included in the reliability analysis, 36% (n=18) were male. The median age was 25 years (range 18-59). The left ankle was involved in 44% (n = 22) of the patients. The mean values of the measurements are given in Table 2.

**Table 2.** MMHA and TCA Measured on Standard Radiographs in Degrees by the Observer 1 (Radiology Resident) and Observer 2 (Orthopaedic Resident).

		MMHA, degrees		TCA, degrees	
		Mean (SD)	Range	Mean (SD)	Range
**Observer 1**	Measurement 1	21.2 (3.2)	13 -28	105.6 (6.1)	93 -118
	Measurement 2	21.1 (3.2)	13 -27	105.9 (6.1)	93 -120
**Observer 2**	Measurement 1	21.5 (3.0)	12 -29	106.8 (5.0)	94 -121

Abbreviations: MMHA, medial malleolar height angle; TCA, talar convexity angle.

The intraobserver reliability for the MMHA and TCA showed excellent ICC values, both exceeding 0.90 (Table 2). The interobserver reliability for the MMHA and TCA was of moderate quality, with ICCs of 0.58 (95% CI 0.37-0.74) and 0.61 (95% CI 0.40-0.77), respectively.

For the MMHA, the MDD indicated a minimal difference in measurements of 1.80 degrees within one observer, and 5.53 degrees between observers, respectively (Table 3, MDD), would be necessary to represent real inpatient variation. For the TCA, the MDD indicated a minimal change of 3.07 degrees (Table 3) within one observer, and 9.84 degrees (Table 3) between observers, respectively, was required to detect a difference.

**Table 3.** Intra- and Interobserver Reliability of the Radiographic Measurements.

	MMHA, degrees	TCA, degrees
Intraobserver		
ICC (95% CI)	0.96 (0.93-0.98)	0.97 (0.94-0.98)
SEM	0.65	1.11
MDD	1.80	3.07
Interobserver		
ICC (95% CI)	0.58 (0.37-0.74)	0.61 (0.40-0.77)
SEM	1.99	3.55
MDD	5.53	9.84

Abbreviations: ICC, intraclass correlation coefficient; MDD, minimal detectable difference; MMHA, medial malleolar height angle; SEM, standard error of measurement; TCA, talar convexity angle.

**Figure 7.**
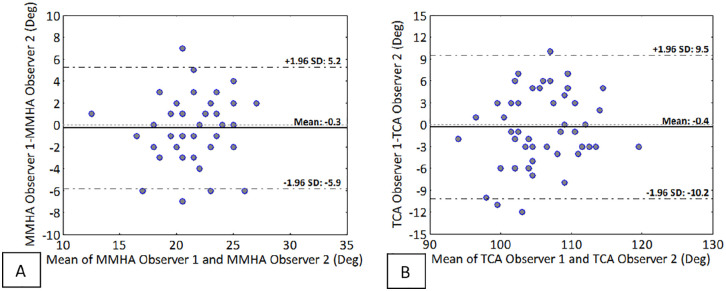
Bland-Altman plots of the interobserver reliability for the (A) medial malleolar height angle (MMHA); (B) talar convexity angle (TCA). Each plot consists of 50 measurements.

The Bland-Altman plots show that the differences in measurements between the observers were not consistent, ranging from –7 to 7 degrees for the MMHA (Figure 7A) and from –12 to 10 degrees for the TCA (Figure 7B). Measurement errors were equally distributed, and 42% (n=21) showed a positive measurement error.

## Discussion

The MMHA and TCA were the result of a translation of 3D talar and tibial shape variations that could be recognized on standard radiographs.^22^ Before evaluating the prognostic value of the 2 angles on the onset of CAI and implementing them in a clinical setting, it is highly important to ensure that the variations of the MMHA and TCA between individuals are not obscured by measurement errors. Therefore, we assessed whether the 2 newly defined angles, MMHA and TCA, can be reliably measured. An additional finding of this study includes that findings in 3D anatomy may be reliably measured on DRR and radiographs if they are assessed in the same mortise view as the lateral radiograph (see Figures 3 and 4).

The translation from 3D anatomy to measurement in 3D space to a measurement on a DRR indicated the measurements had substantial to excellent agreement. The small differences in the subanalyses can be explained by different viewing planes between the angles in 3D space, 2D plane, and overprojection of bony rims on the DRR.

The interobserver reliability was moderate as opposed to the intraobserver reliability. The interobserver reliability value could not be explained by a systematic measurement error, as the consistency in either only a low or high measurement error would lead to a high ICC, which was not observed (see Figure 7). A possible explanation may be that the radiology resident is substantially more experienced in assessing these radiographs and performing specific angle measurements and therefore uses a different technique/interpretation of how to measure the angles. The fact that both measurements suffered from the same problem, an excellent intra- and moderate interobserver reliability, strengthens this explanation.

Despite the variation in measured angles between the observers, the SEM and MDD were relatively low apart from the interobserver MDD of the MMHA.^16,24^ It may be more difficult to define whether a difference can be measured for the MMHA independent of the observers. The MDD of the TCA was relatively small (2.5%-10.5%) indicating that differences that may be clinically relevant can be measured.

Other studies that have taken 3D anatomy into account comparing radiographic alignment measurements with measurements on coronal CT slices indicated substantial to excellent reliability.^3,25^ This study is the first attempt to translate 3D bone shape variations to 2D radiographic measurements based on interpretation of 3D SSMs of the distal tibia and talus, not being dependent on the scanning plane. The main motivation behind this is to strengthen the usability of these models in clinical practice and benefit from their power in identifying shape variations that can hardly be determined with conventional 2D or 3D measurements, and potentially contribute to the development of CAI. Many shape differences have been observed in 3D bone geometry analyses that are not all taken into account. The current study attempted to describe the most notable shape differences and translated these to conventional imaging for use in clinical practice.

This study only takes 2 shape models into regard. Other shape models such as a variation in subtalar anatomy have been identified.^29^ The reason these variations were not included in this study is the great difficulty of assessing the subtalar joint on conventional radiographs, complicating daily clinical assessment. Another aspect that may be regarded as a limitation is the hypothesis both OCDs and CAI share a trauma mechanism and therefore may share bone shapes that allow this trauma mechanism to occur. Furthermore, our study was limited by the fact the 3D models could not be compared to actual radiographs, but only simulations and the radiographs used for the reliability analysis were sometimes of limited quality because of the rotated position of the foot. Not all radiographs were of good quality because of variation in projection or ankle position assumed by patients. Although this increased the difficulty of performing the measurements adequately, it does represent practice. A further concern is the use of nonweightbearing radiographs, especially the influence of those on the identification of point P2 for the TCA measurement. Future studies should compare these values in weightbearing to those from nonweightbearing images.

Despite similarity in the trauma mechanism of OCD and CAI, it is uncertain whether the MMHA and TCA are of prognostic value on the onset of CAI. To gain insight into the relations of the 2 angles with CAI, they could be implemented in a future risk assessment model on CAI. If these parameters prove to be of prognostic value, the greater goal is that a radiologist will be able to measure these angles on conventional radiographs at the first presentation. Patients defined as at risk of CAI may this way qualify for an adjusted rehabilitation protocol or potential early surgical intervention.

In conclusion, based on the similarities in trauma mechanism between OCDs and chronic ankle instability, the height of the medial malleolus and shape of the talus may be indicative of developing CAI. Based on 2 shape models, we defined 2 new radiographic parameters (MMHA and TCA) that are valid compared to the 3D CT data and can be measured with excellent intraobserver reliability. The interobserver reliability on standard radiographic assessment was moderate and indicates training is required to ensure uniformity in measurement technique. The current method may be used to translate more variations in bone shape prior to implementation in clinical practice.
